# Chest Wall Schwannoma: Case Report and a Review of Imaging Findings

**DOI:** 10.7759/cureus.3694

**Published:** 2018-12-05

**Authors:** Aeman Muneeb, Muhammad Salman Khan, Hina Iqbal, Gulnaz Shafqat

**Affiliations:** 1 Radiology, Aga Khan University, Karachi, PAK

**Keywords:** chest wall tumor, neurogenic tumor, schwannoma

## Abstract

A chest wall schwannoma arises from peripheral nerve sheath Schwann cells of the intercostal nerves. We describe the presentation and imaging findings of a patient who presented with a chest wall swelling. The imaging findings were highly suspicious for a chest wall schwannoma and the histopathology confirmed the diagnosis following surgical excision. Imaging findings are reviewed in detail.

## Introduction

A schwannoma is a benign, encapsulated, neurogenic tumor arising from the Schwann cells of the nerve sheath [1]. Most thoracic neurogenic tumors arise in the posterior mediastinum, and a chest wall schwannoma is a rare entity that arises from intercostal nerves [2-3]. They appear as lobulated, well-circumscribed, benign masses and affect men and women equally, mostly between the ages of 20 and 50 years [2,4]. Most benign tumors of the chest wall, including schwannomas, present as slow-growing, painless, palpable masses [5]. Schwannomas are a type of peripheral nerve sheath tumor (PNST) and most commonly occur in spinal nerve roots [5]. The other type of benign PNST is neurofibromas, which are usually identified in superficial nerves [5]. Multiple schwannomas arising from a single peripheral nerve have also been reported [6].

## Case presentation

Presentation

A 39-year-old male reported to the Aga Khan Hospital with the complaint of swelling on the left chest wall for the past three weeks. There was no associated pain or lymphadenopathy. A systemic examination was unremarkable. The patient was referred to radiology for imaging.

Imaging

**Figure 1:**
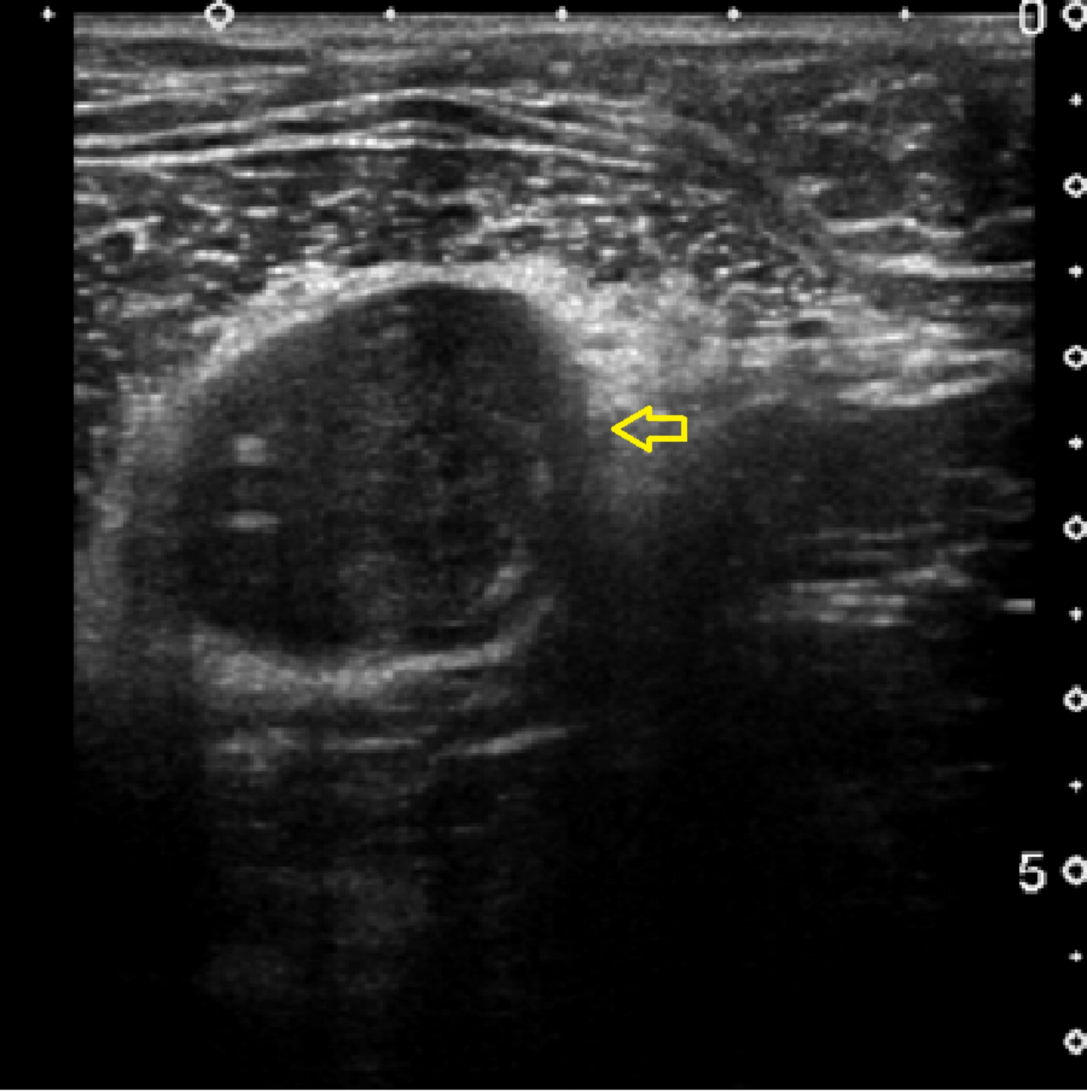
Grayscale ultrasound of chest wall A well-defined hypoechoic solid mass (arrow) noted in between the intermuscular plane showing internal heterogeneity and significant vascularity

**Figure 2:**
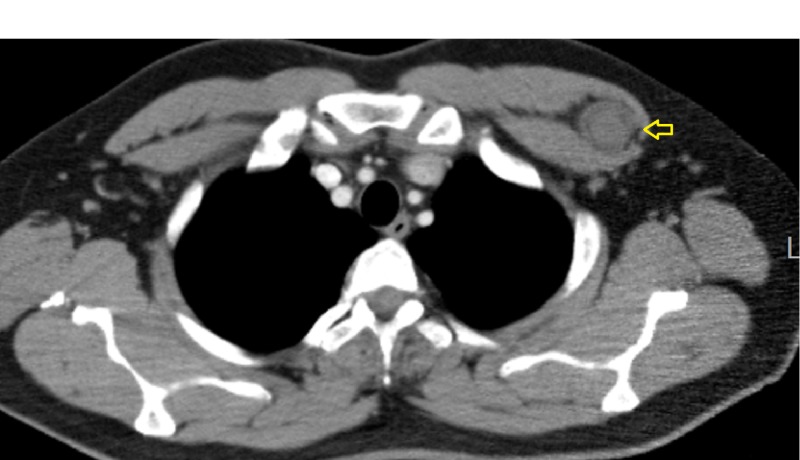
Axial CT of chest (with contrast) Well-defined, rounded, soft tissue density mass (arrow) between the left pectoralis major and minor muscles. CT: computed tomography

**Figure 3:**
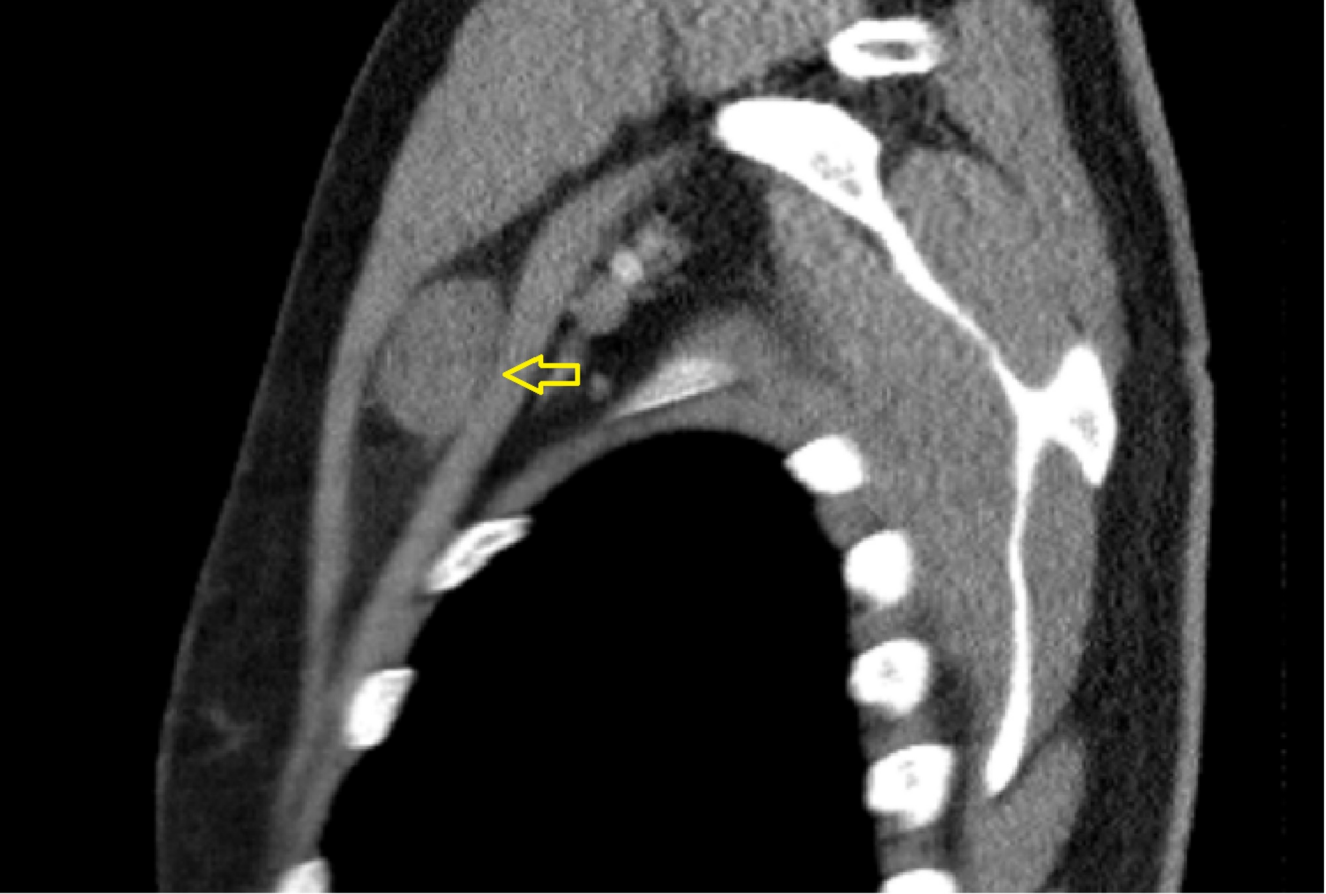
Saggital CT of the chest (with contrast) Redemonstrated well-defined, rounded, soft tissue density (arrow) between the left pectoralis major and minor muscles. CT: computed tomography

**Figure 4:**
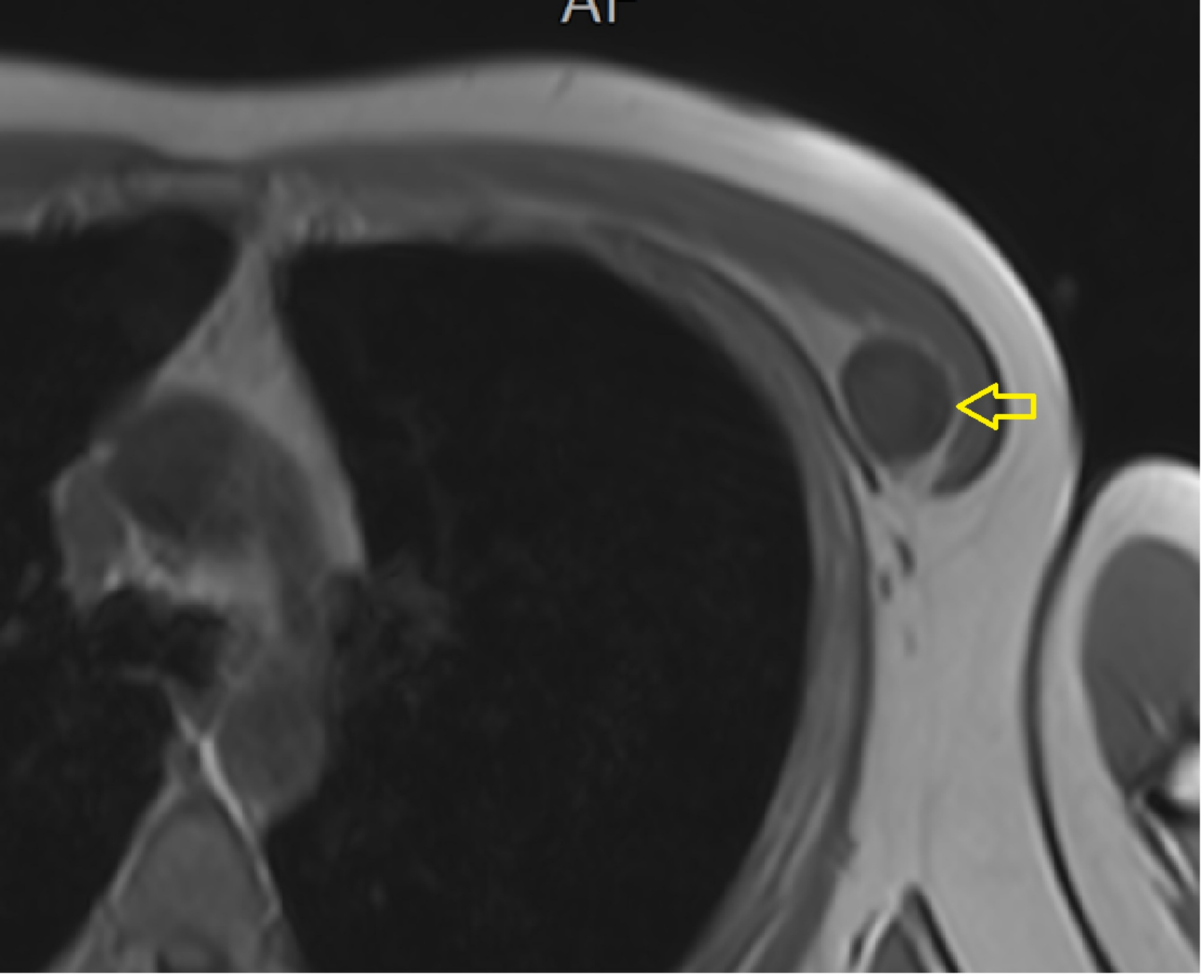
T1-weighted MRI of the chest Isointense, well-defined lesion (arrow) within the intermuscular plane intervening between the left-sided pectoralis major and pectoralis minor muscles. MRI: magnetic resonance imaging

**Figure 5:**
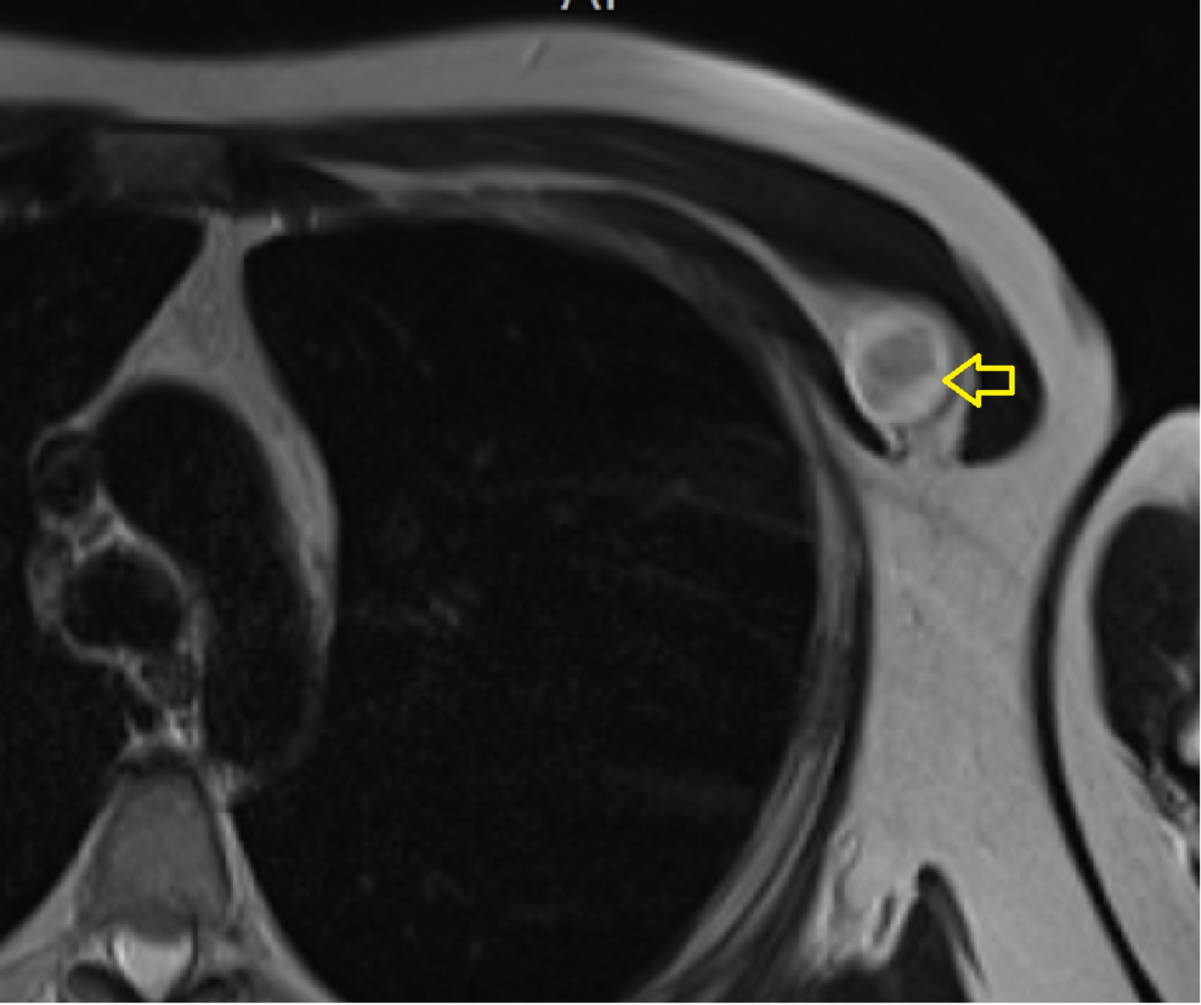
T2-weighted MRI of the chest Heterogeneously hyperintense, well-defined lesion (arrow) within the intermuscular plane, intervening between the left-sided pectoralis major and pectoralis minor muscles. MRI: magnetic resonance imaging

**Figure 6:**
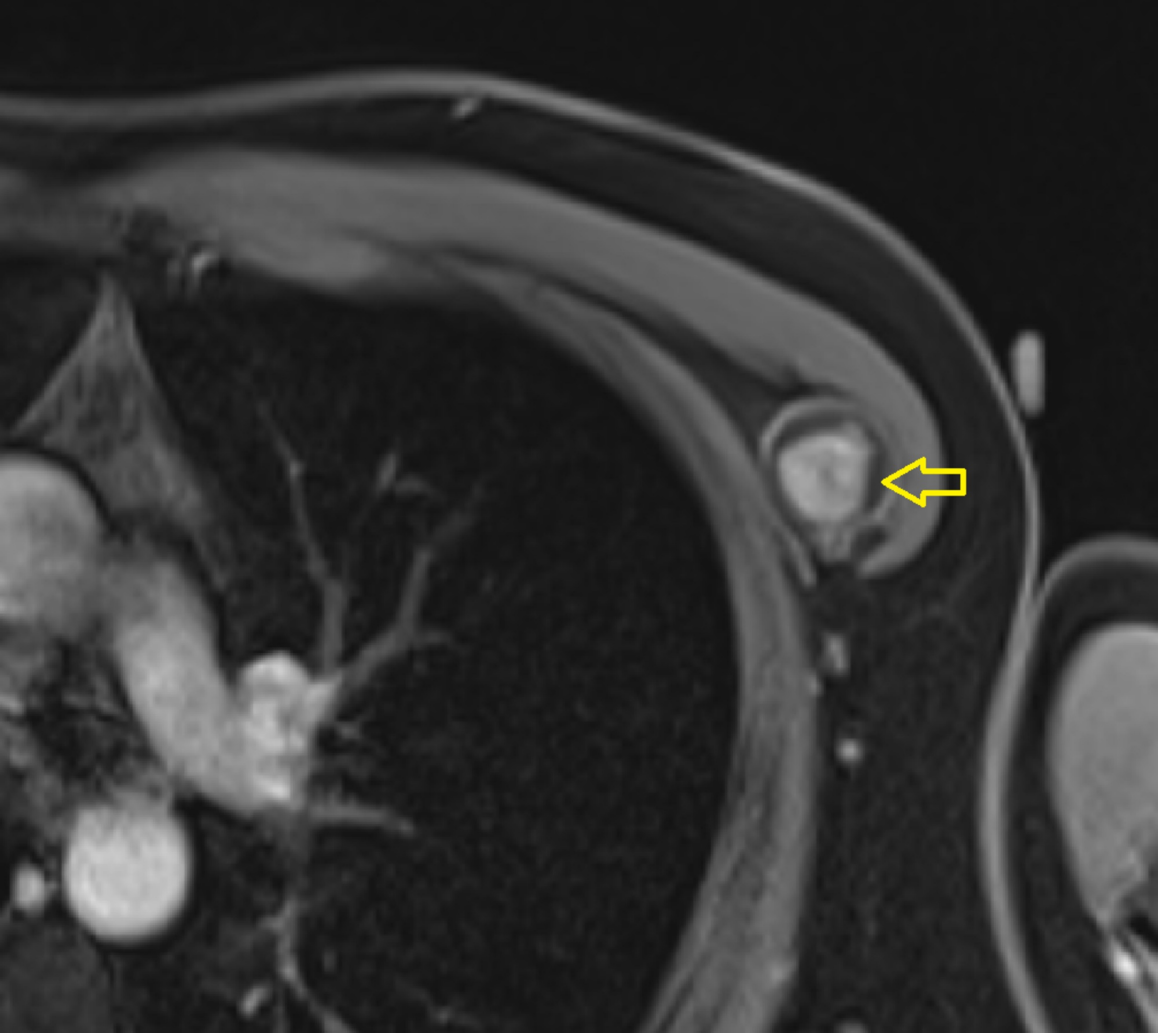
T1-weighted post-contrast MRI Well-defined lesion (arrow) with post-contrast enhancement. MRI: magnetic resonance imaging

An ultrasound of the chest wall demonstrated a well-defined hypoechoic solid mass between the intermuscular plane showing internal heterogeneity and significant vascularity (Figure 1). On computed tomography (CT) imaging, a well-defined, rounded, soft tissue density lesion between the left pectoralis major and minor muscles was seen (Figures 2-3). The magnetic resonance imaging (MRI) scan exhibited a T1 isointense, T2 hyperintense well-defined lesion within the intermuscular plane intervening between the left-sided pectoralis major and pectoralis minor muscles (Figures 4-5). The mass demonstrated post-contrast enhancement (Figure 6).

Management

The patient was admitted and underwent an excision of the chest wall mass under general anesthesia. Post-procedure, the patient was monitored and eventually discharged once stable.

Histopathology

A subsequent histopathological analysis showed a well-demarcated lesion composed of fascicles of spindle cells, exhibiting thick vessels, along with an area showing verrucae bodies. Areas of infarction were also seen. On immunohistochemistry, the tumor was S100 positive. These features were suggestive of a benign neural lesion, most probably a schwannoma.

## Discussion

A chest wall schwannoma is reported to present as a painless mass [5], which is similar to the presentation in our patient. Imaging plays an important role in diagnosis, and non-enhanced CT scans usually demonstrate a homogenous mass with attenuation like that of muscle [4]. On administering contrast, the attenuation of the mass may become slightly more than that of muscle while cystic/necrotic areas will not show any enhancement [4]. Our patient's CT scan similarly demonstrated a well-circumscribed soft tissue density, which is consistent with the findings previously reported in the literature. On MRI, the mass appeared isointense on T1 and hyperintense on T2, which is consistent with the imaging findings reported for schwannomas [4].

The differentials for intramuscular chest wall lesion are glomus tumors and hemangiomas. The CT scans of glomus tumors show a soft-tissue mass with the erosion of adjacent bone. MR images reveal a T1 iso-intense and T2 hyperintense lesion showing post-contrast enhancement. Chest wall hemangiomas are well marginated and have a high signal intensity compared with that of subcutaneous fat on T2-weighted images and iso-intense on T1-weighted images showing post-contrast enhancement [7].

The other differential diagnosis could be a neurofibroma, but these appear target-like on T2 and after contrast administration, the central part appears significantly enhanced [4]. However, the target sign on MRI has also been reported in 0%-54% of schwannomas so while the presence of the target sign points to a peripheral nerve sheath tumor, it does not specifically differentiate between a schwannoma or a neurofibroma [7]. The other differentiating feature is that neurofibromas are usually closely related and invested with the nerve of origin and during excision, the affected nerve needs to be removed as well [7]. From the imaging findings of our case, a peripheral nerve sheath tumor was suspected and histopathology confirmed the final diagnosis. In certain cases, benign chest wall schwannomas may be destructive to the surrounding tissue [8] or may mimic malignant lesions [2]. Malignant transformation has also been reported [5]. Surgical excision is the definitive treatment undertaken to prevent this transformation [5].

## Conclusions

A chest wall schwannoma is a rare tumor arising from peripheral nerve sheath Schwann cells. Previously described imaging criteria can help suspect a PNST and histopathology can be confirmatory. Surgical excision is the mainstay of treatment.
